# Dual role of pyroptosis in liver diseases: mechanisms, implications, and therapeutic perspectives

**DOI:** 10.3389/fcell.2025.1522206

**Published:** 2025-01-23

**Authors:** Siyuan Yang, Yunyi Zou, Chunhua Zhong, Zuoqiong Zhou, Xiyang Peng, Changfa Tang

**Affiliations:** State Key Laboratory of Developmental Biology of Freshwater Fish, Key Laboratory of Physical Fitness and Exercise Rehabilitation of Hunan Province, College of Physical Education, Hunan Normal University, Changsha, China

**Keywords:** pyroptosis, liver disease, NLRP3, inflammation, MAFLD

## Abstract

Pyroptosis, a form of programmed cell death induced by inflammasome with a mechanism distinct from that of apoptosis, occurs via one of the three pathway types: classical, non-classical, and granzyme A/B-dependent pyroptosis pathways. Pyroptosis is implicated in various diseases, notably exhibiting a dual role in liver diseases. It facilitates the clearance of damaged hepatocytes, preventing secondary injury, and triggers immune responses to eliminate pathogens and damaged cells. Conversely, excessive pyroptosis intensifies inflammatory responses, exacerbates hepatocyte damage and promotes the activation and proliferation of hepatic stellate cells, accelerating liver fibrosis. Furthermore, by sustaining an inflammatory state, impacts the survival and proliferation of cancer cells. This review comprehensively summarizes the dual role of pyroptosis in liver diseases and its therapeutic strategies, offering new theoretical foundations and practical guidance for preventing and treating of liver diseases.

## 1 Introduction

Liver disease is a major global health challenge, causing up to two million deaths annually (Devarbhavi et al., 2023). The liver is a highly dynamic metabolic organ crucial for plasma protein synthesis, gluconeogenesis, glycogen storage, cholesterol metabolism, bile acid synthesis, drug/exogenous metabolism, and detoxification (Qian et al., 2021). Maintaining normal liver structure and function requires balancing between cell generation and death across liver tissues. Excessive cell death usually disrupts liver structure and function (Michalopoulos and Bhushan, 2021).

Pyroptosis, an atypical form of programmed cell death, plays a substantial role in the pathogenesis of various liver diseases, such as viral hepatitis, alcoholic liver disease (ALD), metabolic dysfunction-associated steatohepatitis (MASH), drug-induced liver injury (DILI), and hepatocellular carcinoma (HCC). It is mediated by activated cysteine asparaginase (caspase) and gasdermin (GSDM) family proteins. Pyroptosis is characterized by cellular swelling, perforation, membrane rupture, and an intact nucleus, accompanied by the release of inflammatory factors.

Under normal conditions, pyroptosis is a natural immune response that removes pathogens and defective cells from the body. However, excessive activation of cellular pyroptosis may aggravate inflammation, causing cell death and tissue damage. Thus, pyroptosis acts as a double-edged sword and plays various roles in various diseases, particularly liver diseases, where its role has become increasingly prominent, attracting widespread attention from the scientific community. This article reviews the dual role and underlying mechanisms of pyroptosis in liver diseases, providing references and insights for researchers and clinicians in related fields with the hope of advancing the prevention and treatment of liver diseases.

## 2 Pyroptosis

### 2.1 Definitions and history of pyroptosis

Pyroptosis was first identified in 1986 by Friedlander et al., who discovered that exposing primary mouse macrophages to anthrax lethal toxin led to cell death and a rapid expulsion of cellular contents (Friedlander, 1986). This finding initiated the exploration of intricate mechanisms underlying this unique form of cell death. Morphological changes consistent with pyroptosis were first observed in macrophages infected with Gram-negative bacteria in 1992; however, this was mistaken for another form of apoptosis (Fink and Cookson, 2005). In 2001, Boise and Collins named it “pyroptosis,” from the Greek roots *pyro* (fire/heat) and *ptosis* (falling); thus, pyroptosis is a form of programmed inflammatory cell death (D'Souza and Heitman, 2001).

Pyroptosis, analogous to apoptosis, represents a programmed cell death mechanism that encompasses deoxyribonucleic acid (DNA) fragmentation and chromatin nuclear consolidation, ultimately leading to cell death (Kurokawa and Kornbluth, 2009; Fadeel and Orrenius, 2005). Unlike apoptosis, pyroptosis is specifically mediated by the activation of caspase-1, which elicits an inflammatory response. During this process, the N-terminal pore-forming domain of the GSDM protein oligomerizes, forming pores with a diameter of 10–14 nm in the cell membrane. These pores result in the loss of integrity, enabling the secretion of inflammatory factors such as interleukin 1β/interleukin 18 (IL-1β/IL-18) and caspase-1, which have diameters of 4.5 and 7.5 nm, respectively (Ding et al., 2016a); this secretion amplifies the inflammatory response, ultimately causing membrane rupture and cell lysis.

### 2.2 Molecular mechanisms of pyroptosis

The caspase family comprises cysteine proteases that recognize the xxxD sequence of a substrate and cleave the aspartic acid residues, activating the substrate protein. Without an upstream signal, caspases exist in the cytoplasm as inactive zymogens; however, upon receiving an upstream signal, they are activated by self-shearing to recognize and cleave substrates. Based on the classical theory, cell death is primarily caused by inflammasomes—multiprotein complexes that activate caspases to elicit diverse physiological reactions (Wang et al., 2020a). These inflammasomes, comprising fundamental components, protect host cells from endogenous threat signals and invading pathogens. The mechanisms underlying inflammasome-mediated pyroptosis are traditionally dichotomized into classical (activated by caspase-1) and non-classical (activated by caspase-11 in mice or caspase-4/5 in humans) pathways.

#### 2.2.1 Classical inflammasome pathway

The classical inflammasome pathway was first identified in macrophages infected with *Salmonella* as a caspase-1-dependent cell death form (Fink and Cookson, 2006; Hersh et al., 1999). Caspase-1, a vital protein involved in the classical pyroptosis pathway, is activated by the inflammasome, a multimeric complex that is a crucial defense mechanism against infection and an essential component of the natural immune system.

Inflammasomes primarily comprise receptor proteins, apoptosis-associated speak-like proteins containing caspase recruitment domain (CARD) (ASC), and cystatin precursor (pro-caspase-1). Receptor proteins include Toll-like receptor 4 (TLR4) (a transmembrane surface receptor), NOD-like receptor family pyrin domain containing 3 (NLRP3), NOD-like receptor family pyrin domain containing 4 (NLRP4), NOD-like receptor family CARD domain-containing protein 4 (NLRC4) of the NOD-like receptors (NLR) family, Absent in Melanoma 2 (AIM2) of the interferon (IFN)-inducible p200-protein (HIN200) family, and pyrin of the Tripartite motif-containing protein (TRIM) family (Martinon et al., 2002). Most receptor proteins are specifically activated; NLRP1 and NLRPC4 respond only to pathogen-associated molecular patterns (PAMPs), such as cytosolic acyl dipeptides and bacterial flagellin; AIM2 is activated by endogenous pathogen-producing double-stranded DNA; pyrin is activated by pathogenic toxins, such as cytotoxic TcdB; NLRP3 is activated by several factors, encompassing both intracellular stimuli such as reactive oxygen species (ROS), mitochondrial dysfunction, and lysosomal rupture, as well as extracellular signals, like adenosine triphosphate (ATP)-mediated activation of the purinergic receptor type 2 X7 (P2X7), K+ efflux, and Ca2+ influx (Toldo and Abbate, 2018). Furthermore, certain pathogens like *Staphylococcus aureus* and other factors like asbestos (Dostert et al., 2008) and ultraviolet radiation (Feldmeyer et al., 2007) can activate NLRP3 inflammasome.

In response to PAMPs and danger-associated molecular patterns (DAMPs), cytosolic pattern recognition receptors (PRRs, also known as inflammasome sensors) detect stimuli and assemble into inflammasomes through their interactions with ASC and pro-caspase-1. ASC acts as a connector protein, featuring a pyrin domain (PYD) at its N-terminus and a CARD domain at its C-terminus. The PYD domain activates upstream PRRs and prompts the self-aggregation of ASC into dimers (Martinon and Tschopp, 2007). Subsequently, the CARD domain recruits pro-caspase-1, facilitating its binding to the CARD domain of pro-caspase-1. This interaction induces the self-cleavage and activation of adjacent pro-caspase-1 molecules, transforming them from their zymogen state into proteolytic enzymes (Lamkanfi and Dixit, 2014). Activated caspase-1 cleaves the precursor proteins, pro-IL-1β and pro-IL-18, converting them into the mature inflammatory cytokines, IL-1β and IL-18, respectively, which are secreted extracellularly to elicit inflammatory responses. In addition, activated caspase-1 cleaves GSDMD, a crucial component of the GSDM protein family and a key protein in pyroptosis, characterized by the presence of cytotoxic N- and C-terminal inhibitory domains linked by a flexible connector (Zou et al., 2021). Upon cleaving caspase-1 at a specific site within its structural domains, GSDMD oligomerizes, producing a 31-kDa amino-terminal fragment (GSDMD-N). This fragment interacts with membrane lipids such as phosphatidylinositol, phosphatidic acid, and phosphatidylserine on the inner leaflet of cell membranes. Subsequently, GSDMD-N inserts into the lipid bilayer, forming oligomeric pores with an internal diameter ranging from 10 to 14 nm, enabling the release of inflammatory factors, disrupting cellular ion homeostasis, and eventually inducing pyroptosis (Jorquera et al., 2021).

#### 2.2.2 Non-classical pathway

The non-classical pyroptosis pathway involves the direct recognition and binding of lipopolysaccharide (LPS) in the cytoplasm to the N-terminal CARD structural domain of caspase-4/5/11, leading to their activation. LPS, a major component of Gram-negative bacteria, is recognized by the TLR4/MD2/CD14 receptor complex following bacterial entry into the cytoplasm (Ding et al., 2016b; Pilla et al., 2014), which subsequently activates caspase-4/5/11. Next, activated caspase-4/5/11 cleaves GSDMD to generate GSDMD-N, which oligomerizes and forms pores in the cell membrane (Aglietti et al., 2016). This causes K^+^ efflux—a signal that activates NLRP3. Although caspase-4/5/11 cannot cleave pro-IL-18/pro-IL-1β, NLRP3 activation triggers the NLRP3/caspase-1 pathway, driving the maturation and secretion of these cytokines through the GSDMD-N-formed membrane channels, inducing pyroptosis (Shi et al., 2017). Unlike the classical pathway, only caspase-1 cleaves IL-1β and IL-18, whereas other inflammatory caspases handle GSDMD cleavage.

#### 2.2.3 Granzyme A/B-dependent cellular pyroptosis pathway

Granzymes (GZM), a class of homologous serine proteases that share structural and functional similarities, are predominantly expressed in cytotoxic T lymphocytes (CTLs) and natural killer (NK) cells. These enzymes induce cell death by cleaving specific substrates within the target cells. Members of the human GZM family include GZMA, GZMB, GZMH, GZMK, and GZMM. GZM-induced cell death as apoptosis (Hou et al., 2008; Martinvalet et al., 2005). However, a recent study revealed that NK cells and CTLs can induce pyroptosis in GSDMB-expressing cells via cleaving GSDMB by GZMA (Zhou et al., 2020a). Another study showed that CAR-T cells trigger rapid caspase-3 activation in target cells via releasing GZMB, causing caspase-3/GSDME-mediated pyroptosis (Liu et al., 2020a). In addition, GZMB can directly cleave GSDME, inducing pyroptosis. This process enhances the antitumor immune response and effectively suppresses tumor growth (Wang et al., 2017).

### 2.3 Inflammasomes in pyroptosis

#### 2.3.1 NLRP3 inflammasome

The NLRP3 inflammasome is the most well-studied, comprising NLRP3, ASC, and pro-caspase-1, which are coupled through protein interactions. Activation of the NLRP3 inflammasome is associated with various diseases, such as metabolic disorders, multiple sclerosis, and other autoimmune diseases.

NLRP3 inflammasome activation occurs in two signaling steps, namely, initiation and activation. First, exposure to PAMP/DAMP phosphorylates TLR and activates nuclear factor kappa B (NF-κB) signaling, promoting nuclear translocation of NLRP3, pro-IL-1β, and pro-IL-18 (Bauernfeind et al., 2009). This is the first pre-processing step in activating the NLRP3 inflammasome. Subsequently, intracellular and extracellular activation signals activate NLRP3 inflammasome by promoting the oligomerization of inactive NLRP3, ASC, and pro-caspase-1. The NLRP3 inflammasome activation signals confirmed by current studies include.

##### 2.3.1.1 Intracellular and extracellular ionic fluxes

The flow of intracellular and extracellular ions is a factor in NLRP3 inflammasome activation, including K^+^ efflux, Cl^−^ efflux, and altered Ca^2+^ signaling. The roles of various ionic fluxes are as follows:

• K^+^ efflux: As a major upstream factor in NLRP3 activation and inflammasome formation, K^+^ efflux occurs in response to several NLRP3 activators. For example, extracellular ATP activates NLRP3 inflammasome by opening the two-pore structural domains of P2X7 channels and TWIK2 to trigger K^+^ efflux (Rabie et al., 2009; Mariathasan et al., 2006). In addition, K^+^ efflux is induced by Nigerian bacteriocins, short mycopeptides, and pore-forming toxins, which permeabilize the plasma membrane during inflammasome activation (Franchi et al., 2014). Other activators of the NLRP3 inflammasome, including cholesterol crystals and silica, also trigger K+ efflux (Rajamaki et al., 2010).
• Cl^−^ efflux: Cl^−^ channels, including volume-regulated anion channels and chloride-gated intracellular channels (CLICs), regulate NLRP3 inflammasome activation (Tang et al., 2017; Compan et al., 2012; Domingo-Fernandez et al., 2017). During mitochondrial dysfunction, CLICs translocate to the plasma membrane, triggering Cl^−^ efflux, which enhances NLRP3-Nek7 complex formation. However, Cl^−^ efflux alone mediates only ASC binding and oligomerization, which is insufficient for the assembly of functional inflammasome, whereas K^+^ efflux is crucial for the downstream cleavage of caspase-1 and the release of IL-1β.
• Ca^2+^ flow: Artificial removal of intracellular and extracellular Ca^2+^ stores significantly inhibits ASC oligomerization during NLRP3 inflammasome and pro-caspase-1 activation (Franchi et al., 2012). Major intracellular Ca^2+^ sources include the endoplasmic reticulum, which releases Ca^2+^ during stress into the cytoplasm to activate NLRP3 (Wang et al., 2020b). Another source of intracellular Ca^2+^ is lysosomes. Lysosomal damage or dysfunction, a crucial mechanism for activating NLRP3 inflammasome, is often caused by phagocytosis of endogenous particles, such as monosodium urate crystals, cholesterol lipid crystals, deoxy sphingomyelin, and amyloid-β aggregates, as well as exogenous particles, including silica, asbestos, and alum. Phagocytosed crystals accumulate in the lysosomal compartment, causing increased lysosomal acidification, swelling, membrane integrity loss, and rupture to release histone B, a lysosomal enzyme that activates NLRP3 inflammasome (Hornung et al., 2008; Chu et al., 2009; Sheedy et al., 2013).

##### 2.3.1.2 Mitochondria and ROS

Cytoplasmic ROS, generated by reduced nicotinamide adenine dinucleotide phosphate oxidase, is a common activator of the NLRP3 inflammasome, whereas thioredoxin-interacting protein (TXNIP) is an NLRP3 ligand, and under normal conditions, their activity is inhibited when they interact. Physiological ROS levels maintain normal cellular signaling and homeostasis; however, elevated ROS levels disrupt this function, allowing TXNIP binding to NLRP3 and its activation primarily through the leucine-rich repeat (LRR) structural domain (Zhou et al., 2010). Similarly, ROS production initiates signals that activate NLRP3 inflammasome through ROS-dependent transcription factor NF-κB and LPS-mediated deubiquitination of NLRP3 (Juliana et al., 2012).

Mitochondrial DNA (mtDNA), a potent immunostimulant comprising a 16.5-kb double-stranded circular molecule, is highly sensitive to oxidative damage. mtDNA released from the cytoplasm triggers inflammatory responses and responds to NLRP3 activators such as ATP, hexokinase, and Nigerian mycobacteria. Oxidized mtDNA (ox-DNA) directly activates the NLRP3 inflammatory vesicle (Shimada et al., 2012). Furthermore, mitochondrial ROS (mtROS) production is among the first factors believed to activate inflammasome (Heid et al., 2013). Elevated metabolic rates, hypoxic conditions, and membrane damage are among the diverse stress factors that significantly enhance mtROS generation (Sheu, 2019). NLRX1, a conserved NLR protein localized in the mitochondria, promotes mtROS production. Bisphosphatidyl glycerol, a specific phospholipid in the inner mitochondrial membrane, translocates to the outer membrane to bind to the LRRs of NLRP3, activating it. Mitochondria-associated regulatory proteins have also been implicated in NLRP3 inflammasome activation; however, their mechanism of action remains unclear. Both PAMPs and DAMPs stimulate ROS production, inducing the NLRP3 inflammasome assembly.

#### 2.3.2 AIM2 inflammasome

AIM2 is a cytoplasmic protein discovered in 2010 to detect DNA viruses. As an intracytoplasmic DNA receptor, AIM2 comprises an N-terminal pyrin domain with double-stranded (ds) DNA-recognition capability (independent of binding to a specific sequence) and a C-terminal hematopoietic interferon-inducible nuclear (HIN) that recognizes dsDNA (independent of binding to specific sequences). For AIM2 to oligomerize, it must bind at least 80 bp of dsDNA.

Under steady-state conditions, the intramolecular complex formed by the PYD and HIN structural domains of AIM2 remains bound. The PYD, a folded structure with six α-helices belonging to the death domain superfamily, binds specifically to other proteins through PYD-PYD interactions. AIM2 recruits and binds to ASC through this interaction, initiating inflammasome assembly (Lee et al., 2021). When dsDNA from microbial pathogens or damaged host cells bind to the C-terminal HIN structural domain of AIM2, it triggers inflammasome assembly and activates caspase-1, inducing pyroptosis. In addition, the PYD of AIM2 can self-oligomerize to induce its activation (Lu et al., 2014).

## 3 Liver disease and pyroptosis

### 3.1 Viral hepatitis

Viral hepatitis is the most prevalent chronic viral infectious disease globally, characterized by fatal liver inflammation caused by hepatitis virus infection of hepatocytes. Clinical symptoms include anorexia, nausea, upper abdominal discomfort, pain in the liver area, and fatigue. Hepatitis B virus (HBV) (Robinson et al., 2023) and Hepatitis C virus (HCV) (Mohd Hanafiah et al., 2013) are the most common causative agents of liver infections, with an infection rate reaching 90%.

The role of pyroptosis in viral infections remains unclear. Pyroptosis is a pivotal innate immune response within the body, playing a crucial role in counteracting infections and responding to endogenous danger signals. Regarding viral hepatitis, pyroptosis functions as an antiviral defense mechanism by triggering the death of hepatocytes infected with the virus, restricting viral replication and impeding its spread. Over time, HBV has evolved mechanisms to evade the host immune response, and the inflammasome plays a crucial role in suppressing HBV infection *in vivo* (Watashi et al., 2013). Chen et al. detected activated NLRP3 inflammasomes in human peripheral blood mononuclear cells (PBMCs) isolated from patients with acute hepatitis B (Chen et al., 2018a). Weinberger et al. demonstrated that the NLRP3 inflammasome elicits an immune response to the hepatitis B surface antigen (HBsAg) vaccine (Weinberger et al., 2018). In addition, NLRP3-mediated pro-inflammatory cytokine IL-1β significantly inhibits HBV infection (Yu et al., 2017). These findings emphasize the central role of the NLRP3 inflammasome in antiviral defense.

**FIGURE 1:**
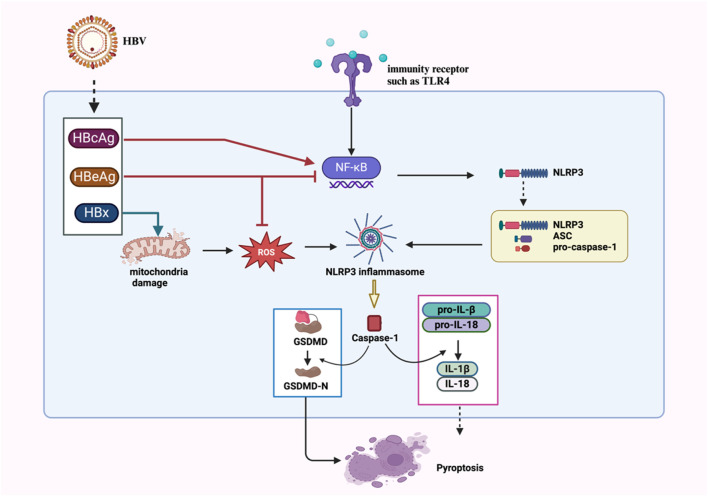
Proposed mechanisms of NLRP3 inflammasome activation and regulation in HBV infection. HBcAg and HBx activate NLRP3 via NF-κB phosphorylation and mtROS pathways, respectively. HBeAg inhibits NLRP3 inflammasome activation via NF-κB signaling pathway and ROS suppression.

Human hepatocytes can increase IL-18 production in response to the hepatitis B virus via upregulating AIM2 inflammasome formation (Pan et al., 2016). Conversely, HBV infection can exploit the regulation of pyroptosis-related inflammasome activation to evade the immune system. HBV-encoded proteins, such as HBp, HBx, HBsAg, and HBeAg, can promote persistent viral infection and immunosuppression by inhibiting innate immune signaling pathways (Bertoletti and Ferrari, 2013). Yu et al. found that HBV suppressed LPS-induced NLRP3 activation in a persistent infection model (Yu et al., 2017). HBeAg partially mediated this inhibitory effect by inhibiting the NF-κB signaling pathway and reducing ROS production. However, Xie et al. discovered that HBx, a key factor in HBV-induced hepatitis, can activate the NLRP3 inflammasome in hepatocytes, increasing mtROS production and promoting pyroptosis (Xie et al., 2020). In addition, HBcAg enhances LPS-induced NLRP3 activation and IL-1β release in HepG2 cells by promoting NF-κB phosphorylation (Ding et al., 2019a). In summary, NLRP3 regulation in HBV infection involves a complex mechanism, with HBx and HBcAg promoting activation and HBeAg inhibiting it (Figure 1).

HCV is a major cause of chronic liver disease. In 2016, researchers first observed pyroptosis in Huh-7.5 cells infected with HCV (JFH-1T) (Kofahi et al., 2016). Further experiments by Kofahi et al. revealed that pyroptosis occurred not only in infected cells and neighboring uninfected cells (Kofahi et al., 2016). In the experiment, the caspase-1-specific inhibitor Z-WEHD-FMK was utilized to treat HCV-infected and control cells. When caspase-1 activity was effectively inhibited, over half of the cells originally destined to die due to HCV infection survived. Additionally, IL-1β was not detected in the supernatants of the infected cells (Kofahi et al., 2016). This discovery confirmed the involvement of pyroptosis in HCV infection. HCV infection is associated with NLRP3 activation, and HCV genomic ribonucleic acid (RNA) reportedly activates NLRP3 in human myeloid cells (Chen et al., 2014). HCV infection of hepatocytes also activates NLRP3, and its over-activation can be inhibited via deubiquitination (Ramachandran et al., 2021a). However, Chen et al. (2014) found that HCV particles do not significantly activate NLRP3 in Huh7 cells and THP-1-derived macrophages (Chen et al., 2014). Negash et al. revealed that the HCV core protein activates NLRP3 via phospholipase C (PLC)-related calcium signaling in liver macrophages, driving IL-1β release and inflammation (Negash et al., 2019a).

HCV glycoprotein can trigger NLRP3 activation and pyroptosis in THP-1 macrophages. Both HCV RNA and proteins can activate NLRP3 under specific conditions, exerting substantial effects on the infection and pathological processes. In addition, HCV-infected cells often exhibit organelle rearrangement (Egger et al., 2002), which may be associated with pyroptosis. Under homeostatic conditions, ASC and IRGM co-localize in the Golgi apparatus. However, HCV infection causes ASC to dissociate from IRGM and the Golgi apparatus and bind to NLRP3. NLRP3 knockdown using small interfering RNA (siRNA) results in reduced Golgi fragmentation, whereas ASC knockdown using siRNA alters the Golgi structure in both control and infected cells and reduces IRGM localization within the Golgi apparatus (Daussy et al., 2021). These findings suggest that ASC regulates IRGM upstream in the pyroptosis pathway and controls the Golgi apparatus. Aggan et al. found elevated serum NLRP3 levels in patients with HCV, which were associated with liver pathological changes, proposing serum NLRP3 levels as a biomarker for hepatic necroinflammatory changes, fibrosis, and steatosis (Aggan et al., 2022). In summary, HCV regulates NLRP3 through multiple pathways, where HCV RNA stimulates TLR7 in endosomes and initiates NLRP3 gene transcription, whereas HCV core protein and virus-induced K^+^ efflux activate NLRP3 inflammasome. These processes, receptors, and complexes play important roles in infection-related diseases and require further investigation.

Currently, interventions targeting pyroptosis for treating viral hepatitis are in the early stages. ATOH8, a basic helix-loop-helix (bHLH) superfamily transcription factor, inhibits hepatocyte pyroptosis and aids HBV immune evasion to interfere with the host’s innate immune system. However, the specific mechanism of its inhibitory action on pyroptosis requires further investigation (Liu et al., 2023). IDN-6556 (Pockros et al., 2007) and PF-03491390 (Shiffman et al., 2010) are potent caspase inhibitors and effective therapeutic agents for patients with HCV. The innate immune restriction factor tetherin (also known as bone marrow stromal cell antigen 2, BST-2), a type II transmembrane protein induced by type I interferons, strongly inhibits HBV-induced pyroptosis. BST-2 may inhibit the HBV progression by blocking the AIM2-dependent signaling pathway. However, further studies are needed to clarify this mechanism (Miyakawa et al., 2015).

### 3.2 Autoimmune hepatitis (AIH)

AIH is a chronic progressive inflammatory liver disease mediated by autoimmune responses and accounts for 10%–20% of chronic hepatitis cases globally, with higher incidence in Europe and North America. It involves an autoimmune response where the immune system mistakenly attacks liver tissues, leading to inflammation and damage (Czaja, 2022). Pyroptosis mediated by inflammasomes is crucial in determining the severity of inflammatory responses and liver damage in AIH.

Hepatocyte death is crucial in AIH progression. In AIH, pyroptosis may lead to the lysis of hepatocytes and the release of inflammatory mediators, which subsequently attract more immune cells to infiltrate the liver, forming a sustained inflammatory cycle. Concanavalin A (ConA)-induced AIH mediates liver injury characterized by T lymphocyte infiltration into the liver, a process triggered by the activation of NK cells and macrophages, leading to cell death and liver damage. Wang et al. reported that pyroptosis is the primary cell death mechanism in mice with AIH, and GSDMD knockout nearly eliminates liver inflammation in mice; this highlights GSDMD-dependent pyroptosis as a key hepatocyte death pathway in AIH (Wang et al., 2022a). Pyroptosis may exacerbate the imbalance of immune regulation by affecting the activation and function of immune cells. This discovery opens new perspectives on the pathogenesis and treatment of AIH.

In addition, the NLRP3 inflammasome plays a substantial role in ConA-induced hepatitis (Guan et al., 2022). In AIH, the activation of inflammasomes is a major driver of exacerbated inflammation and liver fibrosis damage, primarily through initiating pyroptosis and releasing abundant cytokines. This process constitutes a core and crucial event in AIH progression (Beringer and Miossec, 2018). The activation mechanism of NLRP3 may be associated with ROS production in the liver. Hepatitis virus strain-3, heat stress, and CdSe/ZnS quantum dots activate NLRP3 inflammasome in hepatocytes by inducing mtROS (Guo et al., 2015; Lu et al., 2016; Geng et al., 2015). ConA increases ROS production and decreases cellular viability (Zhuang et al., 2016). Furthermore, recombinant human IL-1 receptor antagonists (rHIL-1RAs) alleviate liver inflammation and reduce NLRP3, caspase-1, and IL-1β production in hepatocytes by scavenging ROS and inhibiting pyroptosis (Luan et al., 2018), suggesting that the NLRP3 inflammasome may be a potential therapeutic target for AIH. Notably, although the role of pyroptosis in AIH is increasingly recognized, its specific mechanisms remain incompletely understood. Future studies need to explore the intrinsic connection between pyroptosis and AIH, further to provide new theoretical foundations and practical guidance for preventing and treating the disease.

AIH treatment primarily involves immunoregulatory therapeutic agents, such as glucocorticoids and immunosuppressants. Although immunosuppressive glucocorticoids (for example, prednisone) reduce inflammation, they can have severe side effects. Therefore, using drugs that target pyroptosis and reduce inflammation may represent a novel approach to treating AIH. Dimethyl fumarate (DMF) is a clinically approved fumaric acid derivative used to treat some inflammatory diseases. It reduces mitochondrial damage and mtROS production and enhances the PKA signaling pathway, increasing NLRP3 phosphorylation on Ser/Thr residues at PKA-specific sites and inhibiting NLRP3 inflammasome activation (Shi et al., 2022). DMF inactivates GSDMD through succination and inhibits pyroptosis (Humphries et al., 2020). In addition, DMF can inhibit ASC complex assembly and caspase-1 production to prevent GSDMD-mediated pyroptosis (Shi et al., 2022). Therefore, using DMF could be an effective therapeutic approach for AIH.

Purple sweet potato polysaccharide significantly improves Con A-induced hepatitis by regulating the P2X7R/NLRP3 pathway and reducing oxidative stress (Ding and Fan, 2024). Cucurbitacin E glucoside (CuE), a tetracyclic triterpene glycoside isolated from Cucurbitaceae plants, inhibits oxidative stress by enhancing the SIRT1/Nrf2/HO-1 pathway while suppressing NF-κB/NLRP3 signaling, demonstrating considerable liver protective effects against Con A-induced AIH (Mohamed et al., 2022). Phenethyl isothiocyanate (PEITC), an isothiocyanate compound derived from secondary metabolites of cruciferous plants, inhibits NLRP3 expression and caspase-1 cleavage *in vivo* and *in vitro*. It directly interacts with cysteine 191 of GSDMD to inhibit hepatocyte pyroptosis, positioning PEITC as a new candidate drug for preventing and treating Con A-induced liver injury (Wang et al., 2022b). As mentioned previously, rhIL-1RAs reduce the severity of ConA-induced hepatitis by eliminating ROS, inhibiting NLRP3 inflammasome assembly and activation, preventing pyroptosis, and competing with IL-1β (Wang et al., 2016a). Bone marrow mesenchymal stem cell derived exosomes bind to the 3′UTR of the NLRP3 messenger RNA (mRNA) through exosomal miR-223, which interferes with protein translation. This inhibits NLRP3 inflammasome activation, reversing hepatocyte injury in the S100- or LPS/ATP-induced mouse AIH model (Chen et al., 2018b).

### 3.3 Alcoholic hepatitis (ALD)

Alcoholic hepatitis (ALD) is caused by long-term excessive alcohol consumption. The primary cause of ALD is the direct toxic effects of ethanol and acetaldehyde on the liver. Chronic alcohol abuse can worsen hepatotoxicity due to commonly used medications, certain vitamins, environmental toxins, and carcinogens (Singal et al., 2018). ALD has a high mortality rate, with no effective treatment currently available.

Hepatocyte death following alcohol consumption is a key mechanism in ALD pathogenesis. Several types of cell death, including pyroptosis, occur in cultured hepatocytes and livers of alcohol-exposed rodents, as well as those of patients with ALD (Wang et al., 2016b). Caspase-11 and GSDMD are unregulated in alcohol-exposed mice, with caspase-1 unchanged. In human ALD liver samples, caspase-4 and GSDMD were activated. The caspase-11 knockout mice exhibited reduced GSDMD activation, hepatocyte death, and liver bacterial load, whereas GSDMD overexpression increased hepatocyte death and infiltration of polymorphonuclear leukocytes (PMNs) in the liver. These data support the role of caspase-11/GSDMD in hepatocyte death and PMN infiltration.

Elevated serum IL-18 levels in ALD mouse models and IL-18 knockout mice are proved to be associated with increased liver bacterial load and GSDMD-mediated hepatocyte pyroptosis (Khanova et al., 2018). These results support the role and significance of the caspase-11/4-GSDMD atypical pyroptosis in ALD pathogenesis. Mitochondrial dysfunction plays a crucial role in alcohol-induced hepatocyte regeneration and liver injury. GSDMD downregulation alleviates alcohol-induced pyroptosis and liver injury in ALD mice by improving hepatocyte mitochondrial dysfunction and excessive ROS release (Xie et al., 2024). In addition, studies have confirmed the activation and mechanism of the NLRP3 inflammasome in mice with ALD. Alcohol-induced TXNIP overexpression promotes NLRP3 inflammasome activation and pyroptosis. The primary mechanism involves TXNIP directly interacting with the NLRP3 inflammasome to facilitate the formation of oligomeric complexes (NLRP3/ASC/caspase-1) (Heo et al., 2019),aligning with the findings on activated caspase-1 in hepatocytes of patients with ALD. TXNIP deficiency enhances cell proliferation and resistance to cell death. Furthermore, transfection with miR-148a improves alcohol-induced pyroptosis, suggesting potential therapeutic targets for ALD. Selenium-rich spirulina tablets improve alcohol-induced liver injury by reducing caspase-1-induced pyroptosis (Fu et al., 2018). These data indicate that pyroptosis stimulates and maintains the ALD inflammatory cycle.

Suppressing ROS production can inhibit NLRP3 inflammasome activation and subsequent cellular inflammation. Antioxidant drugs may be potential agents for treating ALD. Quercetin can reduce ROS production, promoting heme oxygenase-1 (HO-1) expression and alleviating acute alcohol-induced liver injury in mice. This, in turn, inhibits NLRP3 inflammasome activation. Therefore, quercetin can counteract alcohol-induced liver injury (Zhao et al., 2022). Oroxylin A, the main active component of *Scutellaria baicalensis*, exhibits multiple pharmacological activities such as anti-inflammatory, antitumor, and vascular protective effects. By reducing ROS accumulation, oroxylin A inhibits NLRP3 inflammasome activation and protects hepatocytes from pyroptosis in alcohol-fed mice via promoting PGC-1α nuclear translocation, increasing Mfn2 transcription, and stabilizing mitochondria (Kai et al., 2020). Diallyl trisulfide, the major organosulfur compound in garlic, reduces the accumulation of intracellular ROS by upregulating hydrogen sulfide levels and inhibiting alcohol-induced NLRP3 inflammasome and GSDMD activation, as well as pyroptosis (Zhu et al., 2022). Sinapic acid (SA, 4-hydroxy-3,5-dimethoxycinnamic acid) is a phenolic acid compound found in various oilseeds, cereals, vegetables, and berries. Bromodomain-containing protein 4 (BRD4) can be considered a therapeutic target for many diseases associated with oxidative stress and pyroptosis. SA treatment significantly abolishes the upregulation of key proteins in the classical pyroptosis pathway in the livers of BRD4 and alcohol-fed mice while enhancing the antioxidant response (Chu et al., 2021).

Dihydroquercetin (TAX), the most abundant dihydroflavonol present in onions, milk thistle, and Douglas fir bark, affects lipid synthesis and oxidation by modulating AMPK activity and reducing alcohol-induced lipid accumulation in mouse livers. TAX inhibits activation of the alcohol-induced P2X7R-Caspase-1-NLRP3 inflammasome and is a potential candidate for treating alcoholic fatty liver disease (Zhang et al., 2018). Cenicriviroc, a novel oral dual CCR2/CCR5 antagonist, prevents alcohol-induced pyroptosis and improves steatohepatitis and liver injury (Ambade et al., 2019). Disulfiram, an alcohol-aversion drug approved by the United States Food and Drug Administration for treating alcohol withdrawal symptoms, inhibits GSDMD-mediated pyroptosis *in vitro* by blocking pore formation and liposome leakage, reducing IL-1β and IL-18 production by activated NLRP3 inflammasomes (Hu et al., 2020).

### 3.4 Metabolic-dysfunction-associated fatty liver disease (MAFLD)

MAFLD, previously known as nonalcoholic fatty liver disease (NAFLD) until a recommendation by an international panel of experts in 2020 to rename it, is a chronic and progressive liver disorder that affects genetically predisposed individuals. This condition arises from a combination of factors, including nutritional excess and insulin resistance. The spectrum of MAFLD encompasses metabolic-associated simple fatty liver, MASH, and MASH-related fibrosis, cirrhosis, and even hepatocellular carcinoma (Eslam et al., 2020). Notably, MASH represents a severe manifestation of MAFLD and serves as an intermediate stage in the progression from simple fatty liver to cirrhosis and liver cancer. With the improvement of living standards globally, the incidence of MAFLD has been increasing annually, and the affected population is becoming younger. Consequently, MAFLD has emerged as the most prevalent chronic liver disease worldwide.

Inflammation, a prominent feature of MAFLD, is linked to pyroptosis and is associated with its progression (Beier and Banales, 2018). GSDMD-N protein levels correlate with the MAFLD Activity Score and fibrosis in patients with MASH. Notably, MAFLD mouse models with GSDMD gene knockout exhibit a significant retardation in liver fibrosis progression, accompanied by reduced release of inflammatory cytokines. Conversely, when GSDMD is overexpressed, this inhibitory effect is completely reversed, generating a large number of cleaved and activated GSDMD-N fragments, which is accompanied by hepatocyte pyroptosis, suggesting its role in MAFLD progression (Xu et al., 2018). It acts by mediating the secretion of pro-inflammatory cytokines (IL-1β, TNF-α, and MCP-1), activating the NF-κB signaling pathway, inducing macrophage infiltration, and enhancing lipogenic gene expression (Xu et al., 2018). Unlike ALD, GSDMD-N elevation in MAFLD is primarily caused by the activation of the NLRP3-ASC-Caspase-1 inflammasome. Therefore, GSDMD is a potential biomarker and therapeutic target for MAFLD (Mai et al., 2020). Gao et al. proposed that estrogen receptor alpha (ERα) can inhibit GSDMD-mediated pyroptosis to improve MAFLD (Gao et al., 2021), suggesting that ERα may be a potential target for NAFLD treatment.

NLRP3 inflammasome activation in the liver promotes MASH progression (Csak et al., 2011). During the development of experimental and clinical MAFLD, NLRP3 expression in the liver is significantly increased (Csak et al., 2014; Szabo and Petrasek, 2015). The pharmacological blockade of NLRP3 can alleviate liver inflammation and fibrosis in experimental MASH models in mice (Mridha et al., 2017). In mouse models with NLRP3, ASC, and caspase-1 knockouts, the hepatocyte death, inflammation, and fibrosis induced by an HFD were all mitigated, further demonstrating the pivotal role of the NLRP3 inflammasome in the pathogenesis of MASH (Wree et al., 2014; Stienstra et al., 2011; Dixon et al., 2013).

Studies have identified multiple mechanisms underlying NLRP3 activation in MAFLD. MAFLD development may be associated with plasma-free fatty acid (FFA) accumulation, particularly palmitic acid, which triggers NLRP3 activation in macrophages via mechanisms involving HIF-1α (Mehal, 2014; Wang et al., 2019; Gupta et al., 2017), cathepsin B (Tang et al., 2018), mitochondrial DNA (Pan et al., 2018), ROS (Yang et al., 2016), and impaired mitophagy flux (Zhang et al., 2019). P2X purinoceptor 7 (P2X7R) plays a crucial role in ATP energy metabolism. Experimental studies have shown elevated P2X7R protein levels in liver biopsy samples from patients with MASH, primarily in macrophages and Kupffer cells, which correlates with increased inflammation and inflammasome activation (Baeza-Raja et al., 2020). Consistent with these findings, P2X7R deficiency reduces NLRP3 inflammasome activation in hepatic sinusoidal endothelial cells and protects mice from liver injury induced by methionine-choline-deficient (MCD) or high-fat diet (HFD) (Blasetti Fantauzzi et al., 2017), demonstrating extracellular ATP involvement in inflammasome activation in MASH.

In Kupffer cells of mice with MASH, interactions between the TXNIP protein and the NLRP3 inflammasome increase. In contrast, mice lacking TXNIP exhibit exacerbated steatosis and liver inflammation when fed an MCD diet (Blasetti Fantauzzi et al., 2017), suggesting a negative regulatory role of TXNIP on NLRP3 and its protective role in MASH. Bile acids regulate inflammasome signaling in hepatocytes by interacting with Takeda-G-protein-receptor-5 (TGR5) or Farnesoid X Receptor (FXR). Reduced TGR5 expression in mice with MASH and human livers correlates with increased NLRP3 activation (Farrell et al., 2019), favoring MASH progression. In addition, FXR deficiency increases NLRP3 activation in the liver (Han et al., 2018), indicating that both TGR5 and FXR inhibit NLRP3 activation in hepatocytes.

In the mouse model of MASH induced through an atherogenic dietary regimen, mice deficient in IL-1β display a notable decrease in liver inflammation and fibrosis compared to their wild-type counterparts (Kamari et al., 2011). The liberation of mitochondrial DNA is a stimulus for NLRP3 activation, subsequently inducing the secretion of IL-1β by Kupffer cells in the context of MASH (Pan et al., 2018). Furthermore, mice with a knockout of the IL-1R1 gene exhibited mitigation of HFD-induced steatosis, inflammation, and fibrotic changes (de Roos et al., 2009). Within hepatocytes, IL-1β is also implicated in facilitating the accumulation of cholesterol and triglyceride. These findings further emphasize the importance of the NLRP3-IL-1β axis in the progression of MAFLD. Although NLRP3 inflammasome activation primarily exhibits detrimental effects in MAFLD, some studies have suggested its potential protective role (Henao-Mejia et al., 2012). However, these views remain controversial, and direct evidence to support them is currently lacking, necessitating further research.

In a mouse model of long-term HFD-induced MASH, AIM2 levels are elevated in the liver (Ganz et al., 2014). When fed a normal diet, AIM2-deficient mice exhibit increased body weight, insulin resistance, and exacerbated adipose tissue inflammation (Gong et al., 2019). This suggests that AIM2 is also involved in MASH progression, with a mechanism that depends on TLR/MyD88 signaling in hepatocytes and macrophages (Lozano-Ruiz and Gonzalez-Navajas, 2020; Csak et al., 2014).

Inhibiting NLRP3 inflammasome activation may represent an important therapeutic approach for MAFLD. Compounds such as actin A (Ruan et al., 2021), Dieckol (Oh et al., 2021), MCC950 (Mridha et al., 2017), and vitamin D (Zhang et al., 2021) suppress pyroptosis, decrease triglyceride and FFA accumulation, improve liver injury, and lower MAFLD scores by inhibiting NLRP3 inflammasome. Taurine alleviates pyroptosis and liver inflammation in an arsenic trioxide (As2O3)-induced MASH model by inhibiting the CTSB-NLRP3 inflammasome pathway (Qiu et al., 2018). Mangiferin upregulates p-AMPKα levels, regulates glucose and lipid metabolism, and downregulates NLRP3 inflammasome-related protein expression, reducing pyroptosis and improving liver injury and other symptoms in mice with MAFLD (Yong et al., 2021). Genipin, an uncoupling protein-2 (UCP2) inhibitor, reverses HFD-induced liver injury, inhibits NLRP3 inflammasome activation, and is associated with UCP2–ROS signaling (Zhong et al., 2018). In addition, long non-coding RNA growth arrest-specific transcript 5 (GAS5) binds to miR-28a-5p in MAFLD, inhibiting NLRP3 inflammasome-mediated hepatocyte pyroptosis (Chen et al., 2023).

Caveolin-1 (Jiang et al., 2023), berberine (Mai et al., 2020), and salvianolic acid A (Ding et al., 2016a) inhibit pyroptosis in MAFLD by suppressing NLRP3 activation mediated by the ROS-TXNIP axis. Liraglutide prevents MASH by blocking NLRP3 inflammasome activation, reducing lipid accumulation, maintaining mitochondrial function, and decreasing ROS production (Yu et al., 2019). Activating the NRF2/HO-1 axis, an important signaling axis against oxidative stress, inhibits NLRP3 inflammasome formation. Baicalein (Shi et al., 2020) and Danshen Zexie decoction (Biao et al., 2022) reduce hepatocyte pyroptosis and protect against HFD-induced inflammation and liver injury through the NRF2/HO-1/NLRP3 pathway.

Exenatide effectively reduces NLRP3, caspase-1, and IL-1β expression in HepG2 cells induced by oleic acid/LPS and in mice with MCD diet-induced liver injury, thus suppressing pyroptosis and alleviating MASH symptoms (Liu et al., 2021). The traditional Chinese medicine formula, Jinlida Granules, decreases NLRP3, caspase-1, IL-1β, and IL-18 expression in HFD-induced mice and FFA-treated HepG2 cells, mitigating liver injury (Hao et al., 2022). The natural plant component, Gardenoside, inhibits pyroptosis-related proteins through the CCCTC-binding factor/dipeptidyl peptidase-4 signaling pathway to improve lipid accumulation and liver fibrosis (Shen et al., 2021). Jiangzhi Ligan decoction exerts hepatoprotective effects in a rat model of HFD-induced MAFLD by regulating the classical and non-classical pyroptosis pathways mediated by GSDMD (Yin et al., 2021).

### 3.5 Primary liver cancer

Primary liver cancer, predominantly HCC, accounts for approximately 90% of liver cancer cases (Petrick et al., 2016) and has high mortality rates globally. Key clinical HCC features include a high recurrence rate and a propensity for metastasis, leading to recurrence in 40%–70% of patients who underwent surgical resection within 5 years (Tampaki et al., 2021). Chemotherapy remains the cornerstone of treating advanced HCC (Grandhi et al., 2016). However, sorafenib, the current standard systemic therapy for patients with advanced HCC, offers relatively limited survival benefits (Liang et al., 2013). Therefore, further exploration of new therapeutic targets and their molecular mechanisms to guide treatment strategies for HCC and improve patient prognosis is crucial.

**FIGURE 2:**
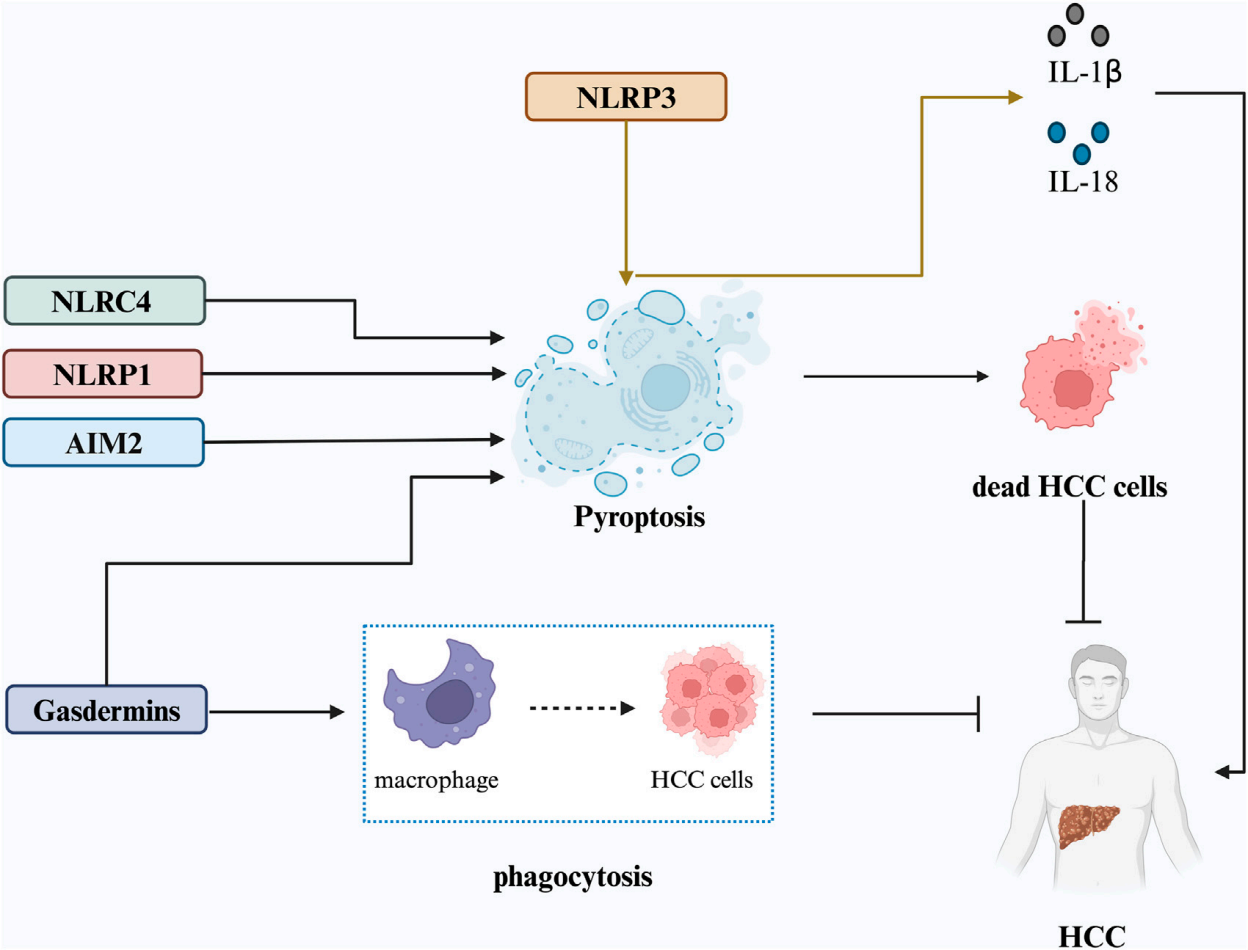
Proposed mechanisms underlying pyroptosis in HCC. Pyroptosis is triggered by inflammasome activation, particularly the NLRP3 inflammasome, leading to the release of IL-1β and IL-18, which fuel the inflammatory cycle in the cancer microenvironment and promote HCC progression. NLRP1, NLRC4, AIM2 inflammasomes, and cleaved gasdermin proteins can also induce pyroptosis, resulting in HCC cell death and inhibition of tumor progression. GSDME expression enhances phagocytosis of tumor cells by tumor-associated macrophages, inhibiting HCC cell growth.

The activation of NLR family proteins and AIM2 is closely associated with HCC progression (Figure 2).NLRP1 activation is favorable for HCC prognosis. Zhou et al. found increased NLRP1 expression in HCC compared to that in normal liver tissue (Zhou et al., 2022). Another study revealed that NLRP1 inflammasome activation leads to caspase-1 activation, IL-1β and IL-18 secretion, and pyroptosis (Grivennikov et al., 2010; Henderson et al., 2021). Furthermore, NLRP1 overexpression is associated with a favorable prognosis in HCC as it leads to immune-mediated tumor eradication and improved prognosis for patients with HCC. However, NLRP3, NLRC4, and caspase-1 overexpression in surrounding non-cancerous tissues is associated with a poorer prognosis postoperatively (Sonohara et al., 2017). NLRP3 inflammasome levels are relatively low in normal hepatocytes but are significantly upregulated in inflammatory liver environments and downregulated in HCC tissues (Wei et al., 2014). IRAK1 downregulation can inhibit the activation of the MAPKs/NLRP3/IL-1β signaling pathway, preventing the proliferation, migration, and invasion of HCC cells (Chen et al., 2020). Anisodamine treatment significantly increases INF-γ and IL-27 levels and decreases TNF-α and IL-4 levels by inhibiting NLRP3, significantly suppressing HCC cell growth (Li et al., 2020). These results suggest that NLRP3 may promote an inflammatory cycle in the cancer microenvironment by mediating the cleavage and release of inflammatory factors such as IL-1β and IL-18, leading to tumor progression. Dead hepatocytes also release DAMPs, causing further inflammatory damage to the liver (Brenner et al., 2013). Furthermore, low AIM2 expression correlates with HCC severity, poor tumor differentiation, and enhanced invasion/metastasis (Chen et al., 2017). Genetic silencing of AIM2 prevents HCC in mice (Martinez-Cardona et al., 2018). Radiofrequency ablation on the proliferation of hepatoma cells is achieved through the induction of pyroptosis via the AIM2 inflammasome signaling pathway (He et al., 2024).

Contrary to the above conclusion, multiple studies have revealed that tumor cells emit danger signals, recruiting antitumor immune cells via pyroptosis. Moreover, these immune cells induce pyroptosis in tumor cells, and the inflammation triggered by pyroptosis elicits a potent antitumor immune response, establishing a positive feedback loop (Wang et al., 2019; Wang et al., 2020c). Wei et al. found that NLRP3 inflammasome reconstitution reverses malignant HCC cells, suggesting that NLRP3 activation may inhibit HCC progression. Specifically, 17β-estradiol (E2) inhibits malignancy in HCC through E2/ERβ/MAPK signaling-mediated upregulation of the NLRP3 inflammasome (Wei et al., 2015). A subsequent study also demonstrated that E2-induced activation of the NLRP3 inflammasome may inhibit HCC progression by triggering pyroptosis (Wei et al., 2019).

Gasdermins are potential new targets for cancer immunotherapy, as they promote antitumor immunity by inducing pyroptosis in tumor cells (Zhang et al., 2020a). Qiu et al. found that GSDMD is a prognostic biomarker and potential therapeutic target for HCC by analyzing its RNA expression, genetic alterations, prognosis, and immune infiltration. GSDMD inhibits tumor proliferation and metastasis by mediating pyroptosis, possibly through a noncanonical pyroptosis pathway (Qiu et al., 2021). Similarly, GSDME is a potential biomarker for HCC diagnosis and prognosis (Hu et al., 2021). GSDME expression enhances phagocytosis of tumor cells by tumor-associated macrophages and increases the number and function of tumor-infiltrating NK and CD8^+^ T lymphocytes. In addition, GSDME promotes tumor suppression by activating pyroptosis and enhancing antitumor immunity (Zhang et al., 2020b).

Pharmacotherapy aimed at inducing pyroptosis in tumor cells is commonly used for cancer treatment. Berberine (Ortiz et al., 2014) is a promising drug for cancer therapy that upregulates caspase-1 mRNA and protein expression in an HCC cell line (HepG2 cells) in a concentration-dependent manner, inhibiting cell survival via caspase-1-mediated pyroptosis (Chu et al., 2016). Miltirone inhibits HCC progression by targeting GSDME-induced pyroptosis, significantly increasing intracellular ROS accumulation while inhibiting MAPK activation and mitogen-activated protein kinase/extracellular signal-regulated kinase phosphorylation, which collectively inhibit the activity of extracellular signal-regulated kinase 1/2 (ERK1/2). This leads to pyroptosis via BAX/caspase-9/caspase-3/GSDME (Zhang et al., 2020a). Cannabidiol induces an integrated stress response and mitochondrial stress in HCC cells, activating ATF4 and its downstream target gene, *CHOP*, leading to upregulated Bax protein expression. This initiates a cellular cascade that culminates in caspase-3/caspase-9/GSDME-mediated pyroptosis (Shangguan et al., 2021).

Chimeric antigen receptor T (CAR-T)-cell immunotherapy has demonstrated efficacy in cancer treatment. CAR-T cells release perforins to form pores that allow GZMB entry into the target tumor cells and caspase-3 activation, leading to GSDME cleavage and pyroptosis (Liu et al., 2020b). The CAR-T cell therapy provides an effective, specific, long-term cancer treatment. In addition, scientists designed NK92 cells expressing a chimeric costimulatory translational receptor (CCCR) comprising the extracellular structural domain of programmed cell death protein 1 (PD1), the transmembrane and cytoplasmic structural domains of NKG2D, and the cytoplasmic structure of 41BB. This receptor converts inhibitory PD1 signaling into activating signaling, effectively enhancing antitumor activity. *In vitro*, CCCR-NK92 cells rapidly kill tumor cells by inducing GSDME-mediated pyroptosis and significantly inhibit tumor growth in a cancer xenograft model (Lu et al., 2020). CCCR-NK92 cells offer a potential “*ex vivo*” immunotherapy for treating PDL1-positive cancers.

Another common cancer treatment strategy involves activating the host immune system to identify and counteract aberrant malignant tumor cells *in vivo*. GZM activation by NK and CTL cells initiates tumor cell pyroptosis. Notably, GSDMB overexpression in HEK-293T cells, which lack endogenous GSDM expression, triggers pyroptosis when co-cultured with human NK cells, independent of caspase involvement (Zhou et al., 2020b). Similarly, perforin, IFN-γ, and several cytokines triggered by immune stimulation can eliminate tumor cells by inducting cellular pyroptosis (Zhang et al., 2020b; Xi et al., 2019).

### 3.6 Drug-induced liver injury (DILI)

The liver is a vital organ for drug aggregation, transformation, and metabolism, and usually, drugs absorbed through the digestive tract usually pass through the portal vein into the liver. DILI refers to liver damage caused by drugs or their metabolites, which may occur owing to hypersensitivity to drugs or decreased tolerance. DILI is a common cause of acute liver failure and poses considerable challenges in clinical practice and drug development (Norman, 2020).

Hepatocyte death is a cause of drug-induced hepatotoxicity and a characteristic feature of DILI. Acetaminophen (APAP), a widely used antipyretic and analgesic, is a hepatotoxin that can induce DILI through a predictable, dose-related mechanism with intrinsic liver injury characteristics (Yan et al., 2018). Earlier studies highlighted the role of NLRP3 inflammasome in APAP-induced DILI. Researchers further elucidated the relative importance of NLRP3 activation in APAP-induced hepatotoxicity by inhibiting inflammasome activity using aspirin or mouse models deficient in NLRP3, ASC, caspase-1, and TLR9 (Imaeda et al., 2009). Wang et al. demonstrated that PRX3 inhibits NLRP3 activation by acting on ROS, mitigating APAP-induced pyroptosis and protecting the liver (Wang et al., 2021). Furthermore, GSDMD enhances the survival capacity of hepatocytes in liver injury induced by acetaminophen. A recent study using GsdmD^−/−^ mice demonstrated that these mice exhibited significantly higher levels of liver injury when exposed to toxic levels of acetaminophen (Yang et al., 2019). In addition, Ouyang et al. observed GSDME activation in APAP-induced DILI mouse models and patient samples and found that GSDME knockout protected mice against APAP-induced pyroptosis and deISGylation of carbamoyl phosphate synthetase-1 (CPS1) while improving drug-induced liver injury (Ouyang et al., 2024). This suggests that GSDME may be a promising therapeutic target for APAP-induced DILI.

The primary treatment strategy for DILI is to discontinue the drug causing the condition immediately (Hassan and Fontana, 2019). Notably, despite drug withdrawal, liver injury often persists in many patients with DILI owing to the activation of the innate immune system and subsequent drug withdrawal-induced cytokine-mediated adaptive immune responses (Mosedale and Watkins, 2017). Therefore, targeting these pathological mechanisms is essential for the complete recovery of patients with DILI. Shikonin, a natural antioxidant and anti-inflammatory compound, can inhibit the hepatotoxicity of APAP by significantly reducing NLRP3 and TLR9 mRNA levels, as well as inflammatory cytokine expression such as IL-1β and IL-6, in APAP-treated mice (Guo et al., 2019). BRB can improve APAP-induced liver injury by inhibiting NLRP3 inflammasome activation and tissue damage, as well as caspase-1 and IL-1β expression, with low-dose BRB showing more pronounced effects (Vivoli et al., 2016). Cisplatin, a widely used chemotherapy drug, can cause hepatotoxicity by increasing inflammatory cytokines (Zhou et al., 2017). Astragaloside IV derived from the traditional Chinese medicine, Huangqi (*Astragalus membranaceus*), exerts cytoprotective effects (Li et al., 2017) and reduces cisplatin-induced liver injury in rats by activating mitophagy to inhibit NLRP3 inflammasome assembly (Qu et al., 2019). Hinokiflavone alleviates APAP-DILI-induced pyroptosis through the SIX4/Akt/Stat3 pathway, showing promise as a potential treatment for DILI (Liu et al., 2024).

## 4 Summary

Cell death under physiological conditions is essential for tissue renewal but can also contribute to disease onset and progression. Recent studies on pyroptosis and liver diseases have confirmed the role of pyroptosis in liver diseases. Mitochondrial dysfunction and excessive ROS production can lead to NF-κB translocation and NLRP3 inflammasome activation, promoting pyroptosis in various liver diseases. Blocking the molecules involved in the pyroptosis pathway (such as NLRP3 and IL-1β) can affect the onset and progression of liver disease, providing potential therapeutic targets for liver disease. However, as pyroptosis is a defense mechanism of the body against pathogens, inhibiting it completely may cause potential adverse effects such as an increased risk of infection. Thus, further studies on disease- or tissue-specific pyroptosis inhibition treatment are warranted.

Cell death under physiological conditions is essential for tissue renewal but contributes to disease onset and progression. Recent studies on pyroptosis and liver diseases have confirmed the role of pyroptosis in liver diseases (Supplementary Table S1). Pyroptosis exhibits a “double-edged sword” effect in liver disease occurrence and development, as it helps eliminate infectious pathogens and abnormal cells and may lead to tissue damage and disease progression when excessively activated. Mitochondrial dysfunction and excessive ROS production can result in NF-κB translocation and NLRP3 inflammasome activation, promoting pyroptosis in various liver diseases. Blocking molecules involved in the pyroptosis pathway can influence the onset and progression of liver diseases, providing potential therapeutic targets for liver diseases. However, since pyroptosis is a defense mechanism of the body against pathogens, completely inhibiting it may cause potential adverse effects, such as an increased risk of infection. Therefore, in the future, it may be necessary to develop personalized and precise pyroptosis targeted therapies for patients with liver disease. By specifically and precisely targeting key molecules in the pyroptosis pathway, such as NLRP3 and GSDMD, pathological processes of liver diseases can be alleviated without compromising the body’s immune defense mechanisms.

In summary, pyroptosis plays a substantial dual role in liver diseases. Future studies should comprehensively consider the pros and cons of pyroptosis and develop effective therapeutic strategies to prevent and control of liver diseases effectively.
