# Supplementary material for: Dual role of pyroptosis in liver diseases: mechanisms, implications, and therapeutic perspectives
Source: Front Cell Dev Biol. 2025 Jan 23;13:1522206. doi: 10.3389/fcell.2025.1522206 (PMC11798966; doi:10.3389/fcell.2025.1522206)
Supplement: Supplementary file 1 [file Table1.docx]

Supplementary Table1. Mechanisms of liver diseases associated with pyroptosis

| Liver Disease | Associated molecular mechanisms of pyroptosis | Reference |
| --- | --- | --- |
| HBV（hbs) | (caspase-1,IL-1β,IL-18)↑ | [1] |
| HBV（HBcAg） | (NLRP3,Caspase-1,IL-1β,IL-18)↑ | [2][3] |
| HBV（HBeAg) | (NF-κB,ROS,NLRP3,IL-1β)↓ | [4] |
| HBV（HBx） | (mtROS,NLRP3,ASC,IL-1β)↑ | [5] |
| HBV | (AIM2,IL-18)↑ | [6] |
| HCV RNA | (ROS,NLRP3,ASC,caspase-1)↑ | [7] |
| HCV | (UCHL5,NLRP3)↑ | [8] |
|  | (Phospholipase-C,Ca2^+^,NLRP3,IL-1β)↑ | [9] |
|  | (ASC-IRGM dissociation,NLRP3)↑ | [10] |
| AIH | (Intestinal barrier,GSDMD) ↑ | [11] |
|  | IL-17 ↑ | [12] |
|  | (ROS,NLRP3)↑ | [13][14][15] |
|  | (NLRP3,IL-1β,caspase-1)↑ | [16] |
| ALD | (caspase11/4,GSDMD)↑ | [17] |
|  | (GSDMD,Mitochondrial dysfunction,ROS,NLRP3,caspase-1)↑ | [18] |
|  | (FoxO1,miR-148a)↓(TXNIP,NLRP3)↑ | [19] |
| MAFLD | (GSDMD,IL-1β,TNF-α,MCP-1,NF-κB,Lipogenesis)↑ | [20] |
|  | (Palmitic acid,NLRP3)↑ | [21][22][23][24][25][26][27] |
|  | (P2RX7,NLRP3,IL-1β)↑ | [28] |
|  | TGR↓,NLRP3↑ | [29] |
|  | FXR↓,NLRP3↑ | [30] |
|  | (MtDNA,NLRP3,IL-1β)↑ | [31] |
|  | (TLR,MyD88,AIM2)↑ | [32][33] |
| HCC | (NLRP1,caspase-1,IL-1β,IL-18)↑ | [34][35] |
|  | NLRP3↓ | [36] |
|  | (IRAK1,MAPKs,NLRP3,IL-1β)↑ | [37] |
|  | (E2,ERβ,MAPK,NLRP3)↑ | [38] |
|  | GSDME↑ | [39] |
| DILI | (Tlr9,pro-IL-1β,pro-IL-18,caspase-1,NLRP3)↑ | [40] |
|  | (MtROS,NLRP3)↑ | [41] |
|  | (ROS,GSDMD,Caspase-8)↑ | [42] |
|  | (CPS1 deISGylation,GSDME)↑ | [43] |

**REFERENCES**

[1] MIYAKAWA K, MATSUNAGA S, WATASHI K, et al. Molecular dissection of HBV evasion from restriction factor tetherin: A new perspective for antiviral cell therapy [J]. Oncotarget, 2015, 6(26): 21840-52.

[2] MANIGOLD T, BOCKER U, CHEN J, et al. Hepatitis B core antigen is a potent inductor of interleukin-18 in peripheral blood mononuclear cells of healthy controls and patients with hepatitis B infection [J]. J Med Virol, 2003, 71(1): 31-40.

[3] DING X, LEI Q, LI T, et al. Hepatitis B core antigen can regulate NLRP3 inflammasome pathway in HepG2 cells [J]. J Med Virol, 2019, 91(8): 1528-36.

[4] YU X, LAN P, HOU X, et al. HBV inhibits LPS-induced NLRP3 inflammasome activation and IL-1beta production via suppressing the NF-kappaB pathway and ROS production [J]. J Hepatol, 2017, 66(4): 693-702.

[5] XIE W H, DING J, XIE X X, et al. Hepatitis B virus X protein promotes liver cell pyroptosis under oxidative stress through NLRP3 inflammasome activation [J]. Inflamm Res, 2020, 69(7): 683-96.

[6] PAN X, XU H, ZHENG C, et al. Human hepatocytes express absent in melanoma 2 and respond to hepatitis B virus with interleukin-18 expression [J]. Virus Genes, 2016, 52(4): 445-52.

[7] CHEN W, XU Y F, LI H, et al. HCV Genomic RNA Activates the NLRP3 Inflammasome in Human Myeloid Cells [J]. Plos One, 2014, 9(1).

[8] RAMACHANDRAN A, KUMAR B, WARIS G, et al. Deubiquitination and Activation of the NLRP3 Inflammasome by UCHL5 in HCV-Infected Cells [J]. Microbiol Spectr, 2021, 9(1): e0075521.

[9] NEGASH A A, OLSON R M, GRIFFIN S, et al. Modulation of calcium signaling pathway by hepatitis C virus core protein stimulates NLRP3 inflammasome activation [J]. Plos Pathog, 2019, 15(2): e1007593.

[10] DAUSSY C F, MONARD S C, GUY C, et al. The Inflammasome Components NLRP3 and ASC Act in Concert with IRGM To Rearrange the Golgi Apparatus during Hepatitis C Virus Infection [J]. J Virol, 2021, 95(3).

[11] WANG K, WU W, JIANG X, et al. Multi-Omics Analysis Reveals the Protection of Gasdermin D in Concanavalin A-Induced Autoimmune Hepatitis [J]. Microbiol Spectr, 2022, 10(5): e0171722.

[12] BERINGER A, MIOSSEC P. IL-17 and IL-17-producing cells and liver diseases, with focus on autoimmune liver diseases [J]. Autoimmun Rev, 2018, 17(12): 1176-85.

[13] GUO S, YANG C, DIAO B, et al. The NLRP3 Inflammasome and IL-1beta Accelerate Immunologically Mediated Pathology in Experimental Viral Fulminant Hepatitis [J]. Plos Pathog, 2015, 11(9): e1005155.

[14] LU Y, XU S, CHEN H, et al. CdSe/ZnS quantum dots induce hepatocyte pyroptosis and liver inflammation via NLRP3 inflammasome activation [J]. Biomaterials, 2016, 90: 27-39.

[15] GENG Y, MA Q, LIU Y N, et al. Heatstroke induces liver injury via IL-1beta and HMGB1-induced pyroptosis [J]. J Hepatol, 2015, 63(3): 622-33.

[16] LUAN J, ZHANG X, WANG S, et al. NOD-Like Receptor Protein 3 Inflammasome-Dependent IL-1beta Accelerated ConA-Induced Hepatitis [J]. Front Immunol, 2018, 9: 758.

[17] KHANOVA E, WU R, WANG W, et al. Pyroptosis by caspase11/4-gasdermin-D pathway in alcoholic hepatitis in mice and patients [J]. Hepatology, 2018, 67(5): 1737-53.

[18] XIE Y, WANG Z, SONG G, et al. GSDMD induces hepatocyte pyroptosis to trigger alcoholic hepatitis through modulating mitochondrial dysfunction [J]. Cell Div, 2024, 19(1): 10.

[20] XU B, JIANG M, CHU Y, et al. Gasdermin D plays a key role as a pyroptosis executor of non-alcoholic steatohepatitis in humans and mice [J]. J Hepatol, 2018, 68(4): 773-82.

[21] MEHAL W Z. The inflammasome in liver injury and non-alcoholic fatty liver disease [J]. Dig Dis, 2014, 32(5): 507-15.

[22] WANG X, DE CARVALHO RIBEIRO M, IRACHETA-VELLVE A, et al. Macrophage-Specific Hypoxia-Inducible Factor-1alpha Contributes to Impaired Autophagic Flux in Nonalcoholic Steatohepatitis [J]. Hepatology, 2019, 69(2): 545-63.

[23] GUPTA N, SAHU A, PRABHAKAR A, et al. Activation of NLRP3 inflammasome complex potentiates venous thrombosis in response to hypoxia [J]. Proc Natl Acad Sci U S A, 2017, 114(18): 4763-8.

[24] TANG Y, CAO G, MIN X, et al. Cathepsin B inhibition ameliorates the non-alcoholic steatohepatitis through suppressing caspase-1 activation [J]. J Physiol Biochem, 2018, 74(4): 503-10.

[25] PAN J, OU Z, CAI C, et al. Fatty acid activates NLRP3 inflammasomes in mouse Kupffer cells through mitochondrial DNA release [J]. Cell Immunol, 2018, 332: 111-20.

[26] YANG G, LEE H E, LEE J Y. A pharmacological inhibitor of NLRP3 inflammasome prevents non-alcoholic fatty liver disease in a mouse model induced by high fat diet [J]. Sci Rep, 2016, 6: 24399.

[27] ZHANG N P, LIU X J, XIE L, et al. Impaired mitophagy triggers NLRP3 inflammasome activation during the progression from nonalcoholic fatty liver to nonalcoholic steatohepatitis [J]. Lab Invest, 2019, 99(6): 749-63.

[28] BAEZA-RAJA B, GOODYEAR A, LIU X, et al. Pharmacological inhibition of P2RX7 ameliorates liver injury by reducing inflammation and fibrosis [J]. Plos One, 2020, 15(6): e0234038.

[29] FARRELL G, SCHATTENBERG J M, LECLERCQ I, et al. Mouse Models of Nonalcoholic Steatohepatitis: Toward Optimization of Their Relevance to Human Nonalcoholic Steatohepatitis [J]. Hepatology, 2019, 69(5): 2241-57.

[30] HAN C Y, RHO H S, KIM A, et al. FXR Inhibits Endoplasmic Reticulum Stress-Induced NLRP3 Inflammasome in Hepatocytes and Ameliorates Liver Injury [J]. Cell Rep, 2018, 24(11): 2985-99.

[31] PAN J, OU Z, CAI C, et al. Fatty acid activates NLRP3 inflammasomes in mouse Kupffer cells through mitochondrial DNA release [J]. Cell Immunol, 2018, 332: 111-20.

[32] CSAK T, PILLAI A, GANZ M, et al. Both bone marrow-derived and non-bone marrow-derived cells contribute to AIM2 and NLRP3 inflammasome activation in a MyD88-dependent manner in dietary steatohepatitis [J]. Liver Int, 2014, 34(9): 1402-13.

[33] LOZANO-RUIZ B, GONZALEZ-NAVAJAS J M. The Emerging Relevance of AIM2 in Liver Disease [J]. Int J Mol Sci, 2020, 21(18).

[34] GRIVENNIKOV S I, GRETEN F R, KARIN M. Immunity, inflammation, and cancer [J]. Cell, 2010, 140(6): 883-99.

[35] HENDERSON J M, XIANG M S W, HUANG J C, et al. Dipeptidyl Peptidase Inhibition Enhances CD8 T Cell Recruitment and Activates Intrahepatic Inflammasome in a Murine Model of Hepatocellular Carcinoma [J]. Cancers (Basel), 2021, 13(21).

[36] WEI Q, MU K, LI T, et al. Deregulation of the NLRP3 inflammasome in hepatic parenchymal cells during liver cancer progression [J]. Lab Invest, 2014, 94(1): 52-62.

[37] CHEN W, WEI T, CHEN Y, et al. Downregulation of IRAK1 Prevents the Malignant Behavior of Hepatocellular Carcinoma Cells by Blocking Activation of the MAPKs/NLRP3/IL-1beta Pathway [J]. Onco Targets Ther, 2020, 13: 12787-96.

[38] WEI Q, GUO P, MU K, et al. Estrogen suppresses hepatocellular carcinoma cells through ERbeta-mediated upregulation of the NLRP3 inflammasome [J]. Lab Invest, 2015, 95(7): 804-16.

[39] ZHANG Z, ZHANG Y, XIA S, et al. Gasdermin E suppresses tumour growth by activating anti-tumour immunity [J]. Nature, 2020, 579(7799): 415-20.

[41] WANG Y, ZHAO Y, WANG Z, et al. Peroxiredoxin 3 Inhibits Acetaminophen-Induced Liver Pyroptosis Through the Regulation of Mitochondrial ROS [J]. Front Immunol, 2021, 12: 652782.

[42] YANG C, SUN P, DENG M, et al. Gasdermin D protects against noninfectious liver injury by regulating apoptosis and necroptosis [J]. Cell Death Dis, 2019, 10(7): 481.

[43] OUYANG S X, ZHU J H, CAO Q, et al. Gasdermin-E-Dependent Non-Canonical Pyroptosis Promotes Drug-Induced Liver Failure by Promoting CPS1 deISGylation and Degradation [J]. Adv Sci (Weinh), 2024, 11(16): e2305715.
